# Commentary: TOpClass (Treatment Optimization and Classification of Perianal Crohn’s Disease) Inaugural Meeting Summary

**DOI:** 10.1016/j.gastha.2026.101114

**Published:** 2026-09-29

**Authors:** Theo Pelly, Luke Hanna, Eathar Shakweh, Shivani Joshi, Phil Tozer, Ailsa Hart, TOpClass Consortium, Easan Anand, Sulak Anandabaskaran, Pooja Datt, Reshma Kanani, Ailsa Hart, Phil Tozer, Kapil Sahnan, Phillip Lung, Gauraang Bhatnagar, Nadia Fathallah, Jeroen Geldof, Isabelle De Kock, Marte Becker, Manon Widenberg, Azucena Salas, Thirza J. Van Croonenburg, Chirag Goyal, Vincent de Parades, Parakkal Deepak, Danny De Looze, Bram Verstockt, Nick Powell, Shaji Sebastian, Laura Hancock, Jordi Rimola, Viviana Parra, Sara Tavares Nogueira, Lilli Lundby, Anders Kirch Dige, Jaap Stoker, Christianne J. Buskens, Stefan D. Holubar, Alyssa Parian, David H. Ballard, Benjamin Cohen, Dermot McGovern, Michael De Gregorio, John Nik Ding, Corina Behrenbruch, Susan J. Connor, Horace RT. Williams, Fabian Juliao Banos, Greg Rosenfeld, Sally Lawrence, David Rubin, Abdul Razack, Jeffrey D. McCurdy, Benjamin McDonald, Amy L. Lightner, Serre-Yu Wong, Victoria Gudiño, Laura Constable, Jalpa Devi, Maria Jose Casanova, Michele Carvello, Antonino Spinelli, Monica Millan, Annalisa Maroli, Amelia Kellar, Michael Johnston, Kristin Johnson, Wojciech Serednicki, Ingrid Ordas

**Affiliations:** 1 Fistula Research Unit, St Mark’s Hospital, London, UK; 2 Department of Metabolism, Digestion and Reproduction, Imperial College London, London, UK

## Introduction

This meeting summary presents the highlights from the inaugural meeting of the Treatment Optimization and Classification of Perianal Crohn’s Disease (TOpClass) consortium, which represents a significant milestone in the efforts of this research collaborative. TOpClass is an international multidisciplinary research consortium established to advance and support global research and collaboration aimed at improving outcomes in perianal Crohn’s disease (pCD). Fistulizing pCD affects approximately 20% of patients with Crohn’s disease (CD)^1^ and remains a poorly understood and clinically morbid manifestation. Advancement in academic research in pCD has been limited by the absence of standardized classification systems, heterogenous disease phenotypes, and a lack of consensus over outcome measures.^2^, ^3^

The meeting was a synchronous hybrid online and in-person event, from June 18 to 20, 2025, in Amersham, England. The primary aim of the meeting was to provide a free educational program to increase understanding of pCD and its multidisciplinary management. The secondary aim was idea generation, collaborative planning, and strategic development to define the consortium’s medium-term research and education agenda.

The work of the TOpClass consortium focuses on pCD, which is a subtype of inflammatory bowel disease (IBD). However, it provides the broader IBD community with a model illustrating the advantages of international collaboration, precise patient phenotyping, and standardized clinical and laboratory data collection, with the potential to yield meaningful improvements in patient outcomes. This manuscript aims to provide a summary of the key topics and an overview of emerging insights from the meetings high-level discussions.

## Meeting Overview

### Day 1: Educational Symposium

The first day of the meeting was a synchronous hybrid (virtual and in-person) educational symposium providing a comprehensive update on pCD, including state-of-the-art consensus guidance, diagnosis, imaging techniques, and interventions. This provided an overview of the diagnosis, assessment, and management of pCD, with several key themes discussed. The program for the educational symposium is outlined in Table 1.

**Table 1:** TOpClass Symposium Agenda

Wednesday June 18			
Session 1: Everything you want to know about assessing perianal Crohn’s disease - How can classifying pCD help me treat my patients? - What causes pCD? - Getting the diagnosis of pCD quickly - Could this be isolated pCD? - How can I avoid missing a fistula cancer? - How to recognize the spectrum of perianal Crohn's lesions (beyond fistulas)	Session 2: Everything you want to know about treating perianal Crohn’s disease - The cost of time: Understanding the impact of delayed diagnosis - Medical treatment of pCD - Surgical treatment of pCD - Panel discussion: Putting it all together	Session 3: What is happening across the globe in perianal Crohn’s disease - Global perianal Crohn’s disease	Session 4: Everything you want to know about managing the different classes of perianal Crohn’s disease - A guide to managing every pCD patient you will ever meet: lessons from the global multidisciplinary team (MDT) - Discussion and closing remarks
Thursday June 19			
Session 1: - Welcome and introduction • Top 10 questions in pCD • Reminder of current TOpClass projects in the pipeline	Session 2: Updates on individual center’s projects - Basic science updates - End points, outcome measures and radiology - Cohorts and trials part 1 Updates on individual center’s works and projects benefiting from consortium support: - Cohorts and trials part 2	Session 3: Future directions - “Big bill” items for future consortium work - TOpClass structure, role, and funding	
Friday June 20			
Collaborative working (parallel session): - Session A: Radiology - Session B: Basic science	Bringing “big bill” items to fruition	Summarizing plans	

#### Key themes

##### Diagnostic delay

The importance of early diagnosis of CD is well established, with diagnostic delay associated with poorer clinical outcomes.^6^ This point was emphasized with regard to perianal CD, in particular, earlier diagnosis of CD in patients presenting with a perianal fistula, to improve long-term patient outcomes. The perianal Red Flag Index (PREFAB, a tool to assist clinicians in diagnosing Crohn’s in patients presenting with perianal fistulae) was discussed as having potential to expedite diagnosis, accelerate access to advanced therapies, and improve clinical outcomes in pCD (Table 2).^5^

**Table 2:** PREFAB and ipCD Criteria

PREFAB criteria – criteria for screening colonoscopy in perianal fistula patients – if ≥ 1 criteria present, refer for Crohn’s disease screening colonoscopy	
Biological suspicion	- Elevated FCP level (≥150 mcg/g)
Clinical suspicion^a^	- Unintentional weight loss - Unexplained diarrhea - Primary sclerosing cholangitis - Ulcerative colitis - >1 internal fistula openings - Fistula involving other organs (vagina/bladder) - Recurrent fistulation (after initial healing) - Proctitis - Anal stenosis
Isolated perianal Crohn’s disease criteria	
Independently diagnostic criteria: If either are present, consider a diagnosis of ipCD	
Histological diagnosis	- Epithelioid granulomata in fistula or surrounding perianal tissue
Macroscopic diagnosis	- Anorectal structuring of ≥1 inflammatory or fissure ulcer evidence on examination
Major and minor diagnostic criteria: If ≥5, consider a diagnosis of ipCD	
Major diagnostic criteria: Score 3	
Major diagnostic criteria	- Advanced fistula complexity (>1 internal opening, >1 discrete fistula, organ fistulation) - Family history of IBD (1st or 2nd relative) Confirmed diagnosis of classic EIM of IBD or orofacial granulomatosis
Minor diagnostic criteria: Score 1	
Minor diagnostic criteria	- Potential, current or previous EIM of IBD (diagnosis unconfirmed) - Suspected oral Crohn’s disease - Suspected genital Crohn’s disease - Coexistent hidradenitis suppurativa - Minor associated perianal disease - Recurrence following fistula repair or lay-open with curative intent

EIM, extra-intestinal manifestation; FCP, faecal calprotectin; PREFAB, prediction tool for early identification of patients at risk of Crohn’s disease in perianal fistulas and abscesses.

a And/or suspicion on imaging if performed.

The isolated perianal Crohn’s disease (ipCD) score was also highlighted. This score provides a structured framework to support clinicians in the consistent diagnosis and management of patients with suspected pCD, potentially enabling earlier identification of this poorly characterized IBD phenotype, and thus patients who may benefit from a trial of anti–tumor necrosis factor (anti-TNF) therapy (Table 2).^4^ The ipCD criteria includes independently diagnostic criteria (the presence of granulomata on histology and the classical ‘Crohn’s perineum’ appearance), as well as major and minor criteria, such as advanced fistula complexity, family history, extra-intestinal manifestations, and oral or genital lesions (Table 2). In patients without independent diagnostic criteria, a score of ≥ 5 is sufficient to consider ipCD, and the use of biologic medications.

##### Nonfistulizing disease

An overview was provided of features of nonfistulizing pCD, including ‘fissures’/ulcers and strictures and how these might be differentiated from non-Crohn’s related lesions. In particular, the identification and classification of nonfistulizing lesions was reviewed. The Cardiff-Hughes classification is available, separating features of pCD into ulceration, fistula/abscess, and stricture, with each feature graded 0–2 in severity.^7^ Its application is somewhat limited by challenges in differentiating fissures and ulcers, as well as defining “gross” skin tags.^7^ Concerning features, such as painful or longstanding ulcers or fissures, warrant biopsy to exclude malignancy. This was followed by an evidence-based review of the management of nonfistulizing pCD. The evidence base for the management of strictures and erosive disease remains limited, and there is a need for future dedicated research to address this knowledge gap. Long-term cohort studies in patients with perianal fissures and ulcers in CD have identified a healing rate of 82% at 3 years, and therefore these lesions are not necessarily progressive over time.^8^ Medical therapy is warranted early in cavitating anal ulcers to avoid complications. Surgical management, particularly relating to skin tags and fissuring disease, should be avoided where possible due to the risk of nonhealing wounds.^9^

##### Applying the TOpClass classification to the management of pCD

The combined medical and surgical management of pCD was reviewed, culminating in an international multidisciplinary panel of experts discussing the optimal management of cases across the full spectrum of pCD including fistulizing disease across the TOpClass classification.^3^ The classification categorizes patients with fistulizing disease into 4 broad classes. Class 1 patients have minimal symptoms. Class 2, chronic symptomatic fistulae, is subdivided into 2a (suitable for repair) 2b (suitable for symptom control), and 2c (requiring defunctioning stoma). Class 3 patients are those following defunctioning stoma who have severe disease with an exhausted perineum and other adverse features and who require proctectomy. Class 4 patients have persistent wounds or sinuses following proctectomy.

Key points regarding the medical management of pCD included the role of dose-adjusted infliximab as the gold standard therapy, with other anti-TNF agents (eg, adalimumab) remaining appropriate first-line options.^10^ Uncertainty persists regarding the comparative efficacy of intravenous vs subcutaneous administration of infliximab. Following anti-TNF failure, second-line options include upadacitinib, ustekinumab, interleukin 23 inhibitors, and vedolizumab; however, the absence of robust comparative effectiveness data to guide sequencing between these agents remains a significant limitation in pCD management. In terms of surgical management, Class 2a patients, with surgically repairable fistulae, should be considered for anatomical repair (with ligation of the intersphincteric fistula tract/advancement flap) or nonanatomical repair (with curettage and internal opening closure procedure).^3^ Class 2b patients, may require examination under anesthesia for palliation, seton insertion ± palliative (p) Video-assisted Anal Fistula Treatment. Rationalization with wide drainage or downstaging (d) Video-assisted Anal Fistula Treatment can be used to simplify the fistula until it is suitable for definitive repair.^3^

### Days 2 and 3: Academic Symposium

The following 2 days provided an opportunity for TOpClass consortium members to share cutting edge research from their centers, in addition to discussing and developing ideas for future research within the framework of an overall strategy. The agenda for the second and third day of the meetings is summarized in Table 2. The academic symposium began with a presentation reporting on the recent James Lind Alliance Priority Setting Partnership “Top Ten research questions” in pCD. These research questions cover a number of unmet needs in pCD, including the need to identify an optimal treatment strategy across the full spectrum of pCD, developing a biological classification, improving quality of life, and reducing the impact of pCD on sexual function, intimacy, and relationships.^11^, ^12^ This was followed by sessions divided into 3 key themes: basic science, end points, and clinical cohorts/trials.

#### Key themes

##### Clinical research

###### Improving and standardizing data collection

The TOpClass consortium consists of approximately 100 members from 20 countries across 5 continents. The heterogenous and complex nature of pCD means that large datasets are necessary to draw significant and generalizable conclusions about the distinct clinical phenotypes outlined in the TOpClass classification. The previous work of the consortium has focused on defining these classes, standardizing definitions to improve the quality of future research, and guiding treatment strategies across the classes.^3^, ^4^, ^5^, ^12^, ^13^

The future direction of the consortium includes building on this work by developing collaborative retrospective and prospective databases, as well as aligning outcome measures and end points of future clinical trials. For future collaborative efforts to be meaningful, data harmonization via an established data dictionary will be crucial to combine existing locally held datasets, as well as to ensure that data is standardized for future prospective consortium (and other) studies.

###### Isolated perianal Crohn’s disease

The TOpClass consortium recently collaborated to establish diagnostic criteria for ipCD, aiming to improve diagnosis and support future research into this underrecognized form of IBD.^4^ Whilst most pCD patients have diagnostic luminal disease, histologic evidence is required in isolated perianal disease, and is seen only 8% of the time in patients with known CD.^4^ Therefore, the majority of patients with ipCD can only be *clinically suspected* (using the new ipCD criteria) at the time of diagnosis. Importantly, discussions emphasized the value of prospective data collection with universal clinical and biological sampling to validate the criteria and to investigate this cohort.

Prospective cohort studies that incorporate long-term outcome data will be essential to determine whether specific features suggestive of ipCD of different ipCD scores are predictive of treatment response to IBD therapies. This insight could refine diagnostic pathways by identifying patients more likely to benefit from Crohn’s medications, ultimately improving outcomes.

###### Optimizing the TOpClass classification for data collection

The TOpClass classification published in 2022 (Table 3),^3^ provides a novel framework for categorizing patients with fistulizing pCD by their clinical phenotype, for clinical and research use. This classification has been validated in retrospective cohorts and will be validated in planned prospective cohorts.^14^ The consortium discussed how the classification could be further optimized and refined in future work, such as subclassification of class 2b patients (eg, those too anatomically complex vs too biologically active for repair).

**Table 3:** The TOpClass Classification

Class	Description
Class 1	Minimal symptoms and anorectal disease burden, requiring minimal intervention
Class 2	Chronic symptomatic fistulae
Class 2a	Symptomatic fistulae suitable for combined medical/surgical closure, where patient goal is also for fistula closure
Class 2b	Chronic symptomatic fistulae unsuitable for surgical repair, where patient’s goal is for symptom control
Class 2c-i	Early, rapidly progressive disease requiring early intervention and consideration of defunctioning stoma
Class 2c-ii	Gradually debilitating symptoms requiring defunctioning ostomy for restoration of QoL
Class 3	Exhausted perineum with irreversible destruction, severely symptomatic despite defunctioning stoma, requires proctectomy
Class 4a	Symptomatic sinuses and wounds following proctectomy that are suitable for repair, and the goal is for repair.
Class 4b	Symptomatic sinuses wounds following proctectomy that are not suitable for repair, and the goal is for symptom control

The consortium identified the need for pan-classification studies to investigate the various classes outlined in the TOpClass classification. These would include RCTs in the Class 2 patients considering both medical and surgical treatments, in particular, highlighting the need to incorporate surgical management into future trials of advanced medical therapies. In the Class 2b, 2c, and 3 groups, there is a need for further investigation of QoL, in particular, regarding the impact of treatments on sexual function and intimacy. The Class 4 group requires foundational consensus work to create a starting point for classification, outcomes and quality of life scoring, as well as biological assessment of this group.

The need for a classification for nonfistulizing pCD to complement the TOpClass classification for fistulizing disease has been previously outlined,^15^ and an expert consensus to develop such a classification has been performed by the consortium,^16^ requiring further validation and integration with the existing fistulizing classification.

### End Points

The key themes of discussion in the end points session identified a number of critical areas for future research. Early discussion focused on magnetic resonance imaging (MRI) volume assessment in fistulizing pCD, which may be a dynamic tool with benefits in the assessment of radiological healing, as well as in patient education, surgical training, and preoperative planning. MRI fistula volume changes consistent with TOpClass classification and assessment of clinical response.^17^, ^18^

MRI protocols for the assessment of fistulizing pCD across centers remain variable, and the need to develop a sequence standard operating procedure (SOP) was discussed. In particular, the use of routine IV contrast remains controversial and benefits from this, such as the ability to differentiate fluid from granulation tissue, do not always translate into changes in surgical decision-making.

The recent TOpClass radiological healing definition was discussed.^12^ A fistula can be defined as radiologically healed by the absence of T2-weighted hyperintense signal in the fistula tract, the absence of contrast enhancement on T1 weighted images (if contrast is used), and a completely fibrotic fistula tract. Challenges in the application of this definition, in particular, how the definition can be applied across a heterogenous patient group in a clinical trial setting will be a focus for future consortium research. Recent work implies that the ‘healed’ definition is more specific than sensitive and may undercall a group of patients who will experience long-term healing despite falling short of it.^19^

Current updates regarding outcome measurement instruments (OMIs) for pCD were discussed in light of the ongoing Core Outcome Measurement Set project. Standardized OMIs are essential in clinical research into pCD to ensure consistency, reliability, and comparability across studies.^20^ The need for novel OMIs to fill gaps around the measurement of incontinence and perineal inflammation as well as the symptomatic response to treatment was discussed, in particular, how these can be developed in parallel with novel MRI-based indices and how they would fit in a treat-to-target strategy.

Future research into Class 4 pCD (patients with poor wound healing and persistent perineal sinus following proctectomy) remains a major unmet need, with significant heterogeneity in the published literature and will require elaboration of existing TOpClass classifications of Class 4a and 4b disease.^4^

### Basic Science

The basic science session identified several challenges associated with tissue sampling in pCD research. The anatomical and pathologic heterogeneity of pCD often makes sampling more technically challenging than in luminal disease. Unlike endoscopic luminal sampling in IBD, samples in pCD are typically obtained during examination under anesthesia by a surgeon. These results in significant variability in the site, method, and type of tissue collected. Given these uncertainties around optimal sampling techniques, the consortium plans to establish SOPs for biological sampling. These SOPs aim to address current inconsistencies and provide guidance for future research. Additionally, the integration of translational substudies into future clinical trials was discussed as a key strategy for identifying biological predictors of treatment response, and enhancing the scientific yield of these studies. The SOPs will be used in the prospective U-Eclipse and Miri-Cure studies, amongst others.

Recent work was discussed relating to the role of calprotectin levels from fistula scrapings as a predictor of disease severity. Fistula scraping calprotectin levels are correlated with the TOpClass classification, as well as to hyperintensity of the fistula tract on MRI imaging levels. Further research is required to investigate this potential biomarker.^21^, ^22^

## Conclusion

The TOpClass inaugural summer meeting achieved its aim of educating a global audience, with over 1200 delegates from more than 80 countries accessing content on the state-of-the-art management of pCD. The scientific meeting laid the foundations for the next wave of research initiatives to expand the understanding of this challenging CD phenotype, with the ultimate goal of improving patient outcomes. More broadly, the emergence of collaborative consortia in IBD, including TOpClass alongside initiatives such as International Organization for the study of Inflammatory Bowel Disease and Stenosis Therapy and Anti-Fibrotic Research, highlights the value of coordinated, multidisciplinary efforts in addressing complex and underrecognized disease phenotypes. Such collaborations offer a powerful framework to focus research priorities, accelerate knowledge generation, and ultimately improve care in difficult-to-treat populations.
